# Epidemiology and outcomes of primary sclerosing cholangitis: an Australian multicentre retrospective cohort study

**DOI:** 10.1007/s12072-022-10356-1

**Published:** 2022-06-03

**Authors:** Natassia Tan, N. Ngu, T. Worland, T. Lee, T. Abrahams, K. Pandya, E. Freeman, N. Hannah, K. Gazelakis, R. G. Madden, K. D. Lynch, Z. Valaydon, S. Sood, A. Dev, S. Bell, A. Thompson, J. Ding, A. J. Nicoll, K. Liu, P. Gow, J. Lubel, W. Kemp, S. K. Roberts, A. Majeed, the Melbourne Liver Group

**Affiliations:** 1 Gastroenterology and Hepatology Department, The Alfred Hospital, 55 Commercial Rd, Melbourne, 3004 Australia; 2 Monash University, Melbourne, Australia; 3 Gastroenterology Department, Monash Health, Melbourne, Australia; 4 Gastroenterology and Hepatology Department, Austin Health, Melbourne, Australia; 5 Gastroenterology and Hepatology Department, St Vincent’s Health, Melbourne, Australia; 6 Gastroenterology and Hepatology Department, Melbourne Health, Melbourne, Australia; 7 Gastroenterology and Hepatology Department, Western Health, Melbourne, Australia; 8 Gastroenterology and Hepatology Department, Royal Adelaide Hospital, Adelaide, Australia; 9 University of Melbourne, Melbourne, Australia; 10 Gastroenterology Department, Eastern Health, Melbourne, Australia; 11 AW Morrow Gastroenterology and Liver Centre, Royal Prince Alfred Hospital, Sydney, Australia

**Keywords:** Primary sclerosing cholangitis, Cirrhosis, Liver transplant, Transplant-free survival, Malignancy, Cholangiocarcinoma, Mortality, Incidence, Relative survival, Risk

## Abstract

**Background and aims:**

Little is known regarding the epidemiology and outcomes of patients with primary sclerosing cholangitis (PSC) in Australia. We, therefore, evaluated the epidemiology and clinical outcomes of PSC in a large cohort of Australian patients and compared these to the general population.

**Methods:**

We conducted a multicentre, retrospective cohort study of PSC patients at nine tertiary liver centers across three Australian states, including two liver transplant centers.

**Results:**

A total of 413 PSC patients with 3,285 person-years of follow-up were included. Three hundred and seventy-one (90%) patients had large duct PSC and 294 (71%) had associated inflammatory bowel disease. A total of 168 (41%) patients developed cirrhosis (including 34 at the time of PSC diagnosis) after a median of 15.8 (95% CI 12.4, NA) years. The composite endpoint of death or liver transplantation occurred in 49 (12%) and 78 (19%) patients, respectively, with a median transplant-free survival of 13.4 (95% CI 12.2–15) years. Compared to the general population, PSC accounted for a 240-fold increased risk of development of cholangiocarcinoma (CCA) and CCA-related death. CCA risk was increased with older age of PSC diagnosis, presence of dominant stricture and colectomy. Compared to same-aged counterparts in the general population, PSC patients who were diagnosed at an older age or with longer disease duration had reduced relative survival.

**Conclusion:**

In this large retrospective cohort study of PSC patients in Australia, increased age and time from diagnosis was associated with increased mortality and morbidity particularly from CCA and development of cirrhosis, necessitating need for liver transplant.

**Supplementary Information:**

The online version contains supplementary material available at 10.1007/s12072-022-10356-1.

## Introduction

Primary sclerosing cholangitis (PSC) is an orphan, cholestatic liver disease characterized by chronic inflammation of the intra- and extra-hepatic biliary tree with multi-focal areas of stricturing [1]. It has a strong association with hepatobiliary cancers and inflammatory bowel disease (IBD) with a heightened risk of colorectal cancer (CRC) in this subgroup [2, 3]. There is no proven effective medical therapy which alters the natural history of PSC [1]. Liver transplantation (LT) is an effective intervention for patients who develop refractory complications or end-stage liver disease; however, disease recurrence can occur in up to 25–30% of grafts [3, 4].

There is a paucity of Australasian literature on PSC, with only two studies published from Australia [5, 6] and one from New Zealand [7]. Two studies highlighted the increased risk of PSC-related outcomes in patients with concomitant IBD [5, 7], but also demonstrated variability in median transplant-free survival. To address the limited Australian epidemiological and outcome data on PSC, we aimed to conduct a multicentre study to accurately describe the epidemiology, clinical characteristics, and outcomes in patients with PSC in Australia. We also described the relative survival of PSC patients compared to the general population.

## Methods

### Study population

We performed a retrospective cohort study of patients diagnosed with PSC from 1st January 2000 to 30th March 2021 at 9 tertiary academic liver centers across Australia in Victoria, New South Wales, and South Australia, including two transplant centers and seven non-transplant tertiary referral centers.

### Inclusion and exclusion criteria

We included all adult patients with a confirmed diagnosis of PSC based on the presence of established cholangiographic or histological features of large or small duct PSC coupled with a cholestatic biochemical profile. Patients with secondary causes of sclerosing cholangitis as per recognized international guidelines were excluded [8–10]. Patients with concomitant liver diseases were not excluded for the purpose of this study.

### Case ascertainment

Case ascertainment was achieved at a local level with a combination of pre-existing or prospectively collected databases in addition to computerized search of patient diagnoses through electronic medical records, radiology, and histology records using the terms—“primary sclerosing cholangitis” and “sclerosing cholangitis”. Pre- and post-liver transplant patient registries were also reviewed at the two transplant centers. All individual medical records were reviewed at a local level to confirm a diagnosis of PSC and accuracy of data collection, including removal of duplicate patient entries when referred for LT.

### Data collection

Clinical demographics and characteristics collected included patient sex, date of birth, date of PSC diagnosis, ethnicity, phenotype, ursodeoxycholic acid (UDCA) use, IBD type, presence of cirrhosis with or without portal hypertension, decompensation events (development of ascites, hepatic encephalopathy, or variceal bleeding), LT indication and date, malignancy type and date of diagnosis.

### Definitions

Evidence for overlap syndrome with autoimmune hepatitis was based on biochemical or serological features with or without histological confirmation. IBD phenotypes of Crohn’s disease, ulcerative colitis or IBD-unclassified (IBDU) were determined via clinical documentation and biochemical, imaging, endoscopic and histological confirmation if available based on consensus guidelines [11]. The presence of cirrhosis and portal hypertension was based on radiological or histological findings if available coupled with supportive clinical, non-invasive testing with transient elastography and laboratory data [12].

### Statistics

Non-parametric continuous variables were presented as medians and interquartile ranges (IQR) and analyzed with the Mann–Whitney *U* test for skewed distributions. Binomial data were presented as percentages and were compared using Chi-square or Fisher’s exact test. The Kaplan Meier method was used to analyze outcomes over time including cirrhosis, malignancy, LT, and death, and to estimate the probability and hazard rate of events over time. Cox proportional hazards model was used to analyze predictors of death or LT, and development of cholangiocarcinoma (CCA) via univariate and multivariate analysis. Patients who were diagnosed with cirrhosis at the time of or within 6 months of PSC diagnosis were not included in the expected survival or hazard rate analysis of cirrhosis.

Standardized incidence (SIR) and mortality ratios (SMR) were calculated with the indirect method using population data from the Australian Institute of Health and Welfare as the reference population. Relative survival is defined as the ratio of the observed all-cause survival among patients and the expected survival in a comparable group in the general population. Life tables for the general Australian population stratified by calendar year, age and sex were used to estimate the relative survival. Observed survival in individuals with PSC was calculated on 2-yearly intervals from the date of PSC diagnosis and compared to age and sex matched data from population life tables as described previously [13].

A two-sided *p*-value of < 0.05 was considered as statistically significant, and 95% confidence intervals are reported where appropriate. Data analysis was performed with SPSS, version 27.0 (IBM) and SAS software, version 9.4 (SAS Institute Inc., Cary, NC, US).

## Results

### Study population

A total of 413 PSC patients were included in the study with a median follow-up time of 7.1 (IQR 3.6–11.7) years, contributing to 3,285 person-years of follow-up time from diagnosis. The median age at PSC diagnosis was 37 (IQR 23–53) years, and 266 (64%) patients were male with the majority (88%) of patients being of European ancestry. Two hundred and nineteen (53%) patients were initially managed at a liver transplant tertiary center. Three hundred and seventy-one (90%) patients had a large duct phenotype at diagnosis, and 294 (71%) had associated IBD. Three (0.7%) patients had concomitant chronic hepatitis C. Two hundred and sixty-four (64%) patients were taking ursodeoxycholic acid (UDCA) at any stage during their follow-up. Further descriptions of the demographics and clinical characteristics of the overall cohort are presented in Table 1.

**Table 1:** Baseline clinical characteristics of the study cohort

	Total
	*N* = 413
Age at diagnosis	37 (23–53)
Male	266 (64.4)
Ethnicity	
European	360 (87.8)
Asian (inc. East Asian)	27 (6.6)
Middle Eastern	17 (4.1)
Hispanic	4 (1.0)
African	2 (0.5)
Transplant center	219 (53)
Phenotype	
Large duct	371 (90.3)
Small duct	40 (9.7)
AIH overlap	58 (14.2)
Concomitant liver disease	
Chronic hepatitis C	3 (0.7%)
Dominant stricture	110 (27.4)
UDCA^a^	264 (63.9)
Liver function at diagnosis	
ALT (IU/L)	93 (51–168)
AST (IU/L)	68 (40–128)
ALP (IU/L)	282 (156–562)
Bilirubin (μmol/L)	16 (9–33)
Albumin (g/L)	37 (33–42)
INR	1.0 (1.0–1.1)
IBD	294 (71.2)
IBD type	
Ulcerative colitis	213 (72.4)
Crohn’s disease	73 (24.8)
IBDU	8 (2.7)

Results presented as median (interquartile range) for continuous variables and absolute numbers (percentages) for categorical variables

*AIH* autoimmune hepatitis, *ALT* alanine aminotransferase, *AST* aspartate aminotransferase, *ALP* alkaline phosphatase, *INR* international normalised ratio, *IBD* inflammatory bowel disease, *IBDU* inflammatory bowel disease unclassified, *UDCA* ursodeoxycholic acid

^a^UDCA use at any stage during data collection

**Fig. 1:**
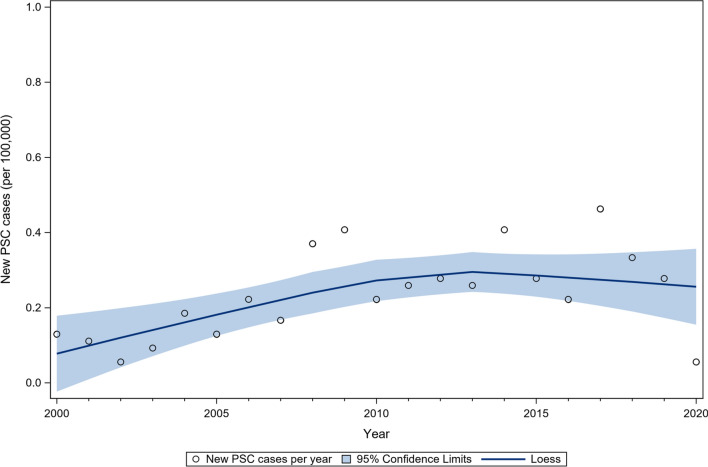
New PSC cases per year in Greater Melbourne

The annual number of new PSC cases at the contributing centers in greater Melbourne increased from 7 in 2000 to a peak of 25 cases in 2017 but declined to 5 toward the end of the study period in 2020/2021. This corresponded to an average annual incidence of 0.27 cases per 100,000 person-year in greater Melbourne (Fig. 1). Towards the end of the study in 2019/2020, there was a total of 266 cases (165 males, 101 females) of PSC alive and managed in the contributing hospitals in greater Melbourne corresponding to an estimated prevalence of 5.9 cases per 100,000 inhabitants (males; 3.7:100,000, females; 2.2:100,000).

### Outcomes

#### Development of cirrhosis

A total of 168 (41%) patients were diagnosed with cirrhosis in our cohort. Thirty-four (8.2%) of patients were found to be cirrhotic at time of PSC diagnosis. Patients with cirrhosis at the time of PSC diagnosis were less likely to have concomitant IBD compared to those who did not have cirrhosis at time of diagnosis (44% vs 74%, *p* < 0.001).

In those who were not cirrhotic at the time of PSC diagnosis, the median time to development of cirrhosis was 15.8 (95% CI 12.4, NA) years (Supp Fig. 1: Cirrhosis-free survival amongst non-cirrhotic patients at diagnosis). The 5-, 10- and 20-year probabilities of cirrhosis were 17.6%, 33.9% and 75.5%, respectively. A total of 135 (80%) cirrhotic patients had concomitant portal hypertension and 82 (49%) experienced a decompensating event.

The incidence rate of cirrhosis in our cohort was 4.9 (95% CI 4.1–5.8) per 100 patient-years. The risk of developing cirrhosis increased with time from PSC diagnosis (Supp Fig. 2: Risk of developing cirrhosis over time). Patients on UDCA were more likely to be diagnosed with cirrhosis (45% vs 33%, *p* = 0.015) but there was no influence on rates of decompensation (22.5% vs 17%, *p* = 0.197) compared to those not on UDCA. There was no difference in time to development of cirrhosis between patients on UDCA and those who were not (*p* = 0.731).

#### Development of malignancy

Fifty-eight (14%) patients developed malignancy after an estimated median of 6.3 (IQR 3.5–10.7) years from diagnosis (Table 2 and 3). Apart from CCA (*n* = 24), CRC (*n* = 12) and hepatocellular carcinoma (HCC) (*n* = 6), four patients were diagnosed with squamous cell carcinoma of the skin, two patients with extranodal marginal zone lymphoma, two patients with neuroendocrine tumors, and one case each of prostate cancer, post-transplant lymphoproliferative disorder, ovarian cancer, breast cancer, non-small cell lung cancer and an appendiceal mucinous neoplasm. Two patients had metastatic malignancy of unclear primary. There was no difference in UDCA use between patients who developed malignancy and those who did not. There was a higher proportion of cirrhotic patients (55.2% vs 44.8%, *p* = 0.021) in patients who developed malignancy.

**Table 2:** Observed outcomes in the study cohort

	Total
	*N* = 413
Cirrhosis	168 (40.7)
Cirrhosis at diagnosis	34 (8.2)
Portal hypertension	135 (32.6)
Hepatic decompensation	82 (19.8)
LT	78 (18.9)
End-stage liver disease	62 (83.8)
HCC	3 (4.1)
Recurrent cholangitis	5 (6.8)
Refractory pruritus	1 (1.4)
CCA	1 (1.4)
Other	2 (2.7)
Death	49 (11.9)
End-stage liver disease	12 (31.6)
CCA	15 (39.5)
CRC	1 (2.6)
HCC	1 (2.6)
Other malignancy	5 (13.2)
Cholangitis	1 (2.6)
Other	3 (7.9)
Malignancy	58 (14)
HCC	6 (1.5)
CRC	12 (2.9)
CCA	24 (5.8)
Others	16 (3.8)

Results presented as absolute numbers (percentages) for categorical variables

*LT* liver transplantation, *HCC* hepatocellular carcinoma, *CCA* cholangiocarcinoma, *CRC* colorectal carcinoma

**Table 3:** Malignant outcomes in the PSC cohort

Malignancy	Observed events	Time (years) to malignancy median (IQR)	Annual risk (%)	Expected events	SIR (95% CI)	*p*-value	Follow-up time from cancer (P-Yr)	Cancer-related death	SMR (95% CI)	*p*-value
CCA	24	6.7 (4.5–11.0)	0.74	0.1	243.6 (146.2–341.1)	0.0001	205.7	15	242.1 (119.6–364.5)	0.0001
HCC	6	6.3 (5.5–11.1)	0.18	0.17	35.4 (7.1–63.7)	0.02	80.7	1	6.9 (0–20.4)	0.39
CRC	10^a^	7.1 (4.9–9.7)	0.31	1.95	5.1 (1.9–8.3)	0.01	133.8	1	1.3 (0–3.8)	0.82

*CCA* cholangiocarcinoma, *CRC* colorectal cancer, *CI* confidence interval, *HCC* hepatocellular carcinoma, *P-Yr* patient-years, *SIR* standardized incidence ratio, *SMR* standardized mortality ratio

^a^Two cases not included in analysis as date of cancer diagnosis not obtained

*Cholangiocarcinoma* CCA was the most common malignancy diagnosed with 24 (5.8%) patients developing CCA after a median of 6.7 (IQR 4.5–11.0) years from PSC diagnosis. Two patients were diagnosed within the first year of PSC diagnosis. Two others were diagnosed in liver explant (4- and 8-year post-PSC diagnosis). The median age at CCA diagnosis was 52.5 (IQR 38–65) years, and the majority (70%) were males. PSC was associated with over 240-fold increased risk for CCA compared to the general population (Table 3), with the highest risk for CCA observed 7–11 years after the diagnosis of PSC (Supp Fig. 3: Risk of developing malignancy after PSC diagnosis). CCA in PSC was also associated with an over 240-fold risk for death with a median survival of only 7 months (Supp Fig. 4: Overall survival after diagnosis of CCA—all-cause mortality). On Cox proportional hazards multivariate analysis, the presence of dominant stricture (HR 3.33, 95% CI 1.40–7.88), history of colectomy (HR 2.95, 95% CI 1.29–6.76) and increased age at PSC diagnosis (HR 1.03, 95% CI 1.00–1.05) were independently associated with an increased risk of CCA (Supplementary Table 2).

*Colorectal carcinoma* Twelve (2.9%) patients developed colorectal carcinoma (CRC) after a median of 7.1 (IQR 4.9–9.7) years from PSC diagnosis. Ten (83%) of those who developed CRC had a pre-existing diagnosis of IBD. PSC was associated with a fivefold increased risk for CRC as compared with the general population, with the highest risk between 6 and 8 years after PSC diagnosis (Supp Fig. 3: Risk of developing malignancy after PSC diagnosis). However, only one patient died of CRC and observed mortality from CRC in PSC patients (Table 3) was comparable to that of CRC in the general population (SMR 1.3, 95% CI 0–3.8, *p*-value 0.82). One patient was diagnosed initially with CRC and subsequently CCA 7 years after.

*Hepatocellular carcinoma* Six (1.5%) patients developed hepatocellular carcinoma (HCC), all of whom were cirrhotic, after a median time from PSC diagnosis of 6.3 (5.5–11.1) years. One HCC-related death was observed in the cohort during a median follow-up of 4.3 (IQR 2.1–7.6) years after HCC diagnosis. PSC was associated with a 35-fold increased risk for HCC (Table 3), but comparable HCC-related mortality to that of the general population (SMR 6.9, 95% CI 0–20.4, *p*-value 0.39).

### Survival after PSC diagnosis

#### Transplant-free survival

**Fig. 2:**
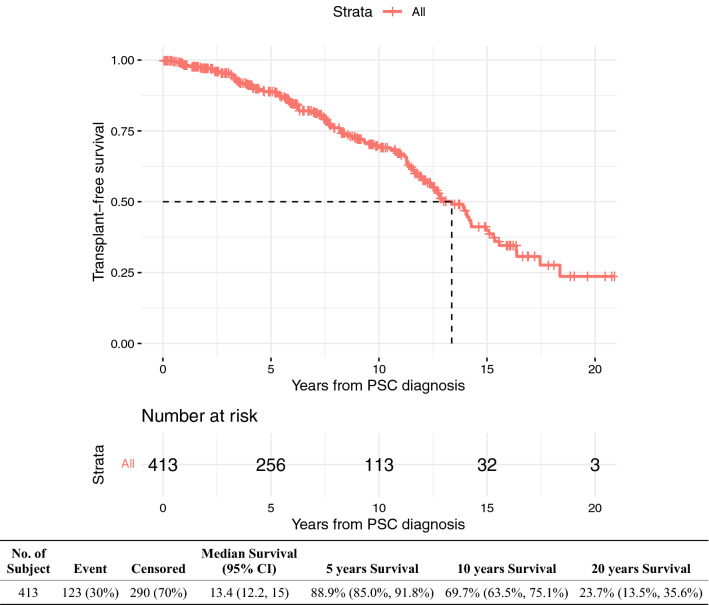
Kaplan Meier survival curve—transplant-free survival

One hundred and twenty-three (30%) patients died or received a LT after an estimated median of 13.4 (95% CI 12.2–15) years from PSC diagnosis (Table 2, Fig. 2). The 5-, 10- and 20-year transplant-free survival rates were 88.9%, 69.7% and 23.7%, respectively. Patients on UDCA were more likely to have died (14.4% vs 7.4%, *p* = 0.03) but there was no difference in need for LT (20.5% vs 16.1%, *p* = 0.28).

The presence of cirrhosis, dominant stricture, CCA, increased age, alkaline phosphatase (ALP) and bilirubin levels at diagnosis were significant on univariate analysis for reduced transplant-free survival. After adjusting for the above variables, only cirrhosis (HR 6.49, 95% CI 2.90–14.6, *p* < 0.0001), CCA (HR 3.03, 95% CI 1.28–7.15, *p* = 0.011) and increased age at PSC diagnosis (HR 1.02, 95% CI 1.01–1.03, *p* = 0.003) were associated with reduced transplant-free survival on multivariate analysis (Supplementary Table 3).

#### All-cause mortality

Forty-nine (12%) patients died during the follow-up period. Median survival was unable to be estimated with Kaplan Meier analysis (Supp Fig. 5: All-cause mortality in the PSC cohort), and mean estimated survival was 17.6 (95% CI 16.8–18.5) years. The 5-, 10- and 20-year survival rates were 95.2%, 89.6% and 60.3%, respectively. The most common cause of death was CCA (*n* = 15, 39.5%), followed by end-stage liver disease (*n* = 12, 32%) and other malignancy in five (13%) patients. Further details of causes of death are illustrated in Table 2.

#### Liver transplantation

Seventy-eight (19%) patients received a LT during the follow-up period. Median time to LT was unable to be estimated with Kaplan Meier analysis (Supp Fig. 6: Cumulative risk of liver transplantation after PSC diagnosis), and mean time to LT was 15 (95% CI 14.1–15.9) years. The most common indication for LT was end-stage liver disease in 62 (84%) patients. LT for malignancy was performed in three (4%) patients for HCC and one (1%) for CCA. Further details of indications for LT in the cohort are illustrated in Table 2.

#### Relative survival compared to the general population

**Fig. 3:**
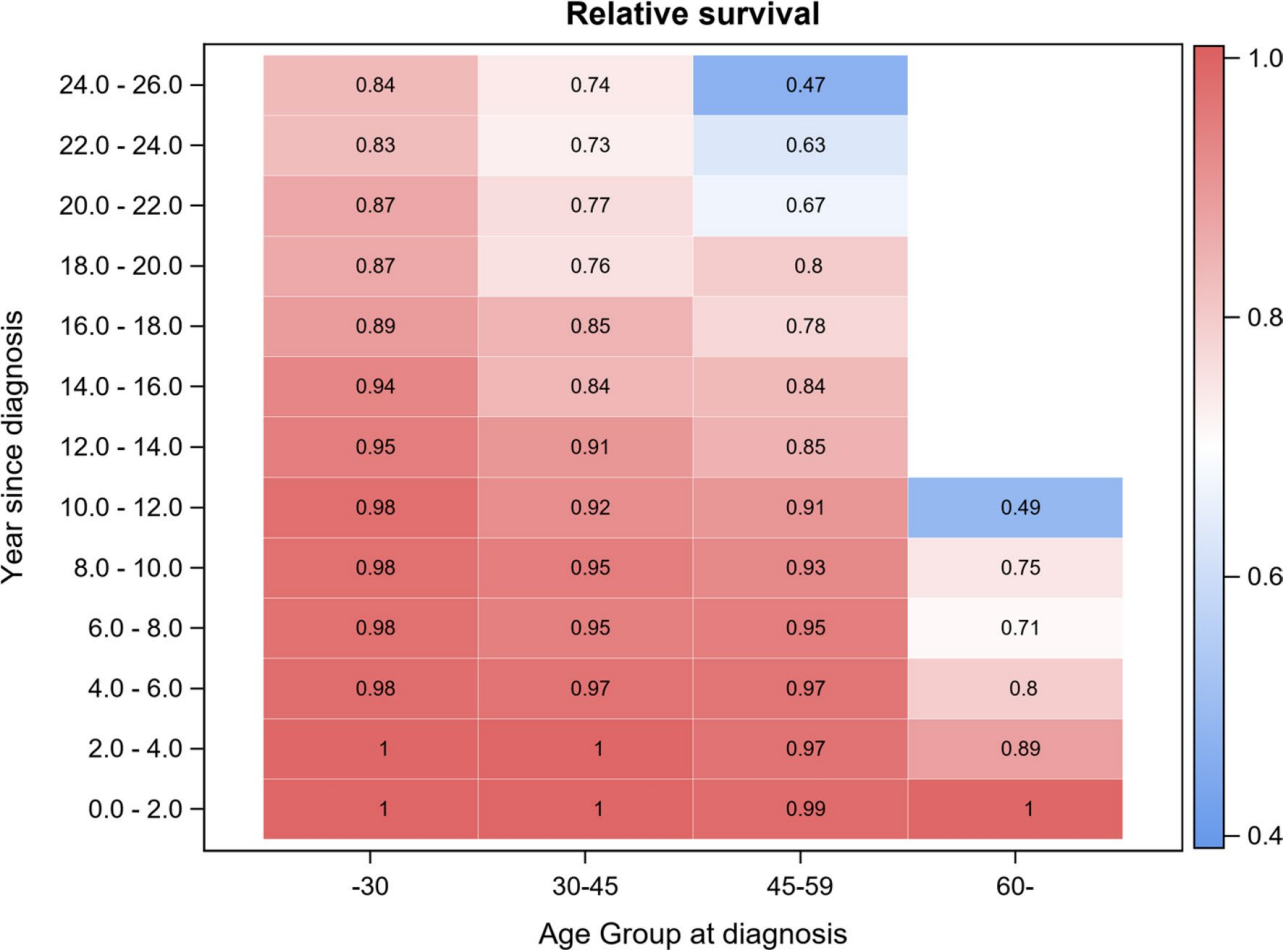
Relative survival according to age and time since PSC diagnosis

Relative survival of PSC patients compared to the Australian general population as stratified by the age of PSC diagnosis is presented in Supplementary Table 1 and Fig. 3. Patients in all age groups had comparable survival to the general population at time of PSC diagnosis; however, there was a trend towards reduced relative survival in PSC patients with increased time from diagnosis (Fig. 3). This reduction in relative survival was steeper and occurred sooner after diagnosis in patients who were diagnosed at older ages, especially in patients diagnosed above 60 years of age (Supplementary Table 1). Survival in patients diagnosed with PSC before the age of 30 dropped below that of the general population first after 12–14 years from PSC diagnosis. The corresponding time point for those diagnosed with PSC between the age of 30 and 59 years was 6–8 years after PSC diagnosis. For those aged above 60 at the time of PSC diagnosis, survival was lower than the general populations by 2–4 years after PSC diagnosis.

## Discussion

### Key findings

This is the largest published study of a Southern Hemisphere cohort of patients with PSC and demonstrates that the natural history of PSC patients in Australia is similar to that of their Northern Hemisphere counterparts. PSC remains an uncommon disease in Australia (266 patients in 2019/2020 managed in 7 Victorian hospitals with a catchment population of ~ 4.5 million). Thirty percent of the cohort required liver transplant or died at a median time from diagnosis of 13 years, with development of cirrhosis, CCA and increased age at PSC diagnosis being significantly associated with reduced transplant-free survival. Despite the increased risk of developing hepatobiliary and colorectal malignancy, only PSC-related CCA was associated with an over 240-fold increase in mortality compared to the general population with a median survival of only 7 months. The presence of a dominant stricture, history of colectomy and increased age at PSC diagnosis was significantly associated with increased risk of CCA development. Furthermore, we demonstrate a reduction in relative survival with increased age and time from diagnosis as compared to the Australian general population.

### Comparison to previous studies

Our cohort draws similarities to other European populations with the majority being of male gender with concomitant IBD and a median age of diagnosis at 37 years [3, 14–16]. Our estimated prevalence rate of 5.9 cases per 100,000 inhabitants is similar to the highest prevalence reported in a United Kingdom study [15]; however, this was significantly lower than the point prevalence of 11 cases per 100,000 inhabitants in Canterbury, New Zealand [7]. This difference in prevalence despite geographical proximity may be due to complete identification of all PSC patients under the care of a small group of gastroenterologists in Canterbury, reflecting a true population prevalence rate [7]. An alternative explanation would be an increased burden of PSC in association with predisposition towards autoimmune disease at higher latitudes [17].

Forty-one percent of our study cohort were cirrhotic, with 8% diagnosed with cirrhosis at time of PSC diagnosis. Patients who were cirrhotic at diagnosis were less likely to have concomitant IBD, which may reflect regular screening with liver function tests prompting earlier diagnosis in this cohort. Majority of cirrhotic patients had concomitant portal hypertension and almost 50% experienced at least one episode of hepatic decompensation. This seems to be higher compared to other cholestatic liver conditions such as primary biliary cholangitis, where less than 30% of patients develop liver failure 10 years after diagnosis [18]. Perhaps related to the lack of an effective medical therapy, PSC patients seem to develop cirrhosis and liver decompensation at higher rates compared to other liver conditions. The finding of increased proportion of cirrhosis in patients on UDCA therapy likely reflects a degree of selection bias rather than true influence of UDCA on outcomes, with patients of a more severe phenotype and/or higher ALP levels being more likely to be commenced on therapy. This may also explain the increased proportion of deaths in patients who took UDCA compared to those who did not.

The proportion of PSC patients that develop cirrhosis has been reported to develop at an incidence rate of 18.6 per 1000 person-years in a American population-based study [15], which is significantly lower than our incidence rate of 4 per 100 person-years. This may reflect an element of referral bias, as half of our patients were managed primarily at transplant centers. Also, the increased risk of developing cirrhosis early after diagnosis (Supp Fig. 2: Risk of developing cirrhosis over time) may reflect latent disease that had been undiagnosed for years in asymptomatic patients or be representative of patients with an aggressive disease phenotype.

CCA remains a significant cause of morbidity and mortality in PSC patients [2, 19, 20]. The proportion of patients who developed CCA (5.8%) is comparable to other studies with prevalence ranging from 2.5 to 12% [2, 14, 21, 22]. The median time from diagnosis to development of CCA in our cohort was 6.7 years, which mirrors the findings of a large population-based study in the Netherlands where median time to CCA was 6 years [20]. This is longer compared to previous studies where most cases of CCA were detected in the first year of PSC diagnosis [14, 20, 22]. The risk of CCA and CRC development compared to the general population are similar to other European cohorts at 240-fold and fivefold, respectively [14, 20]. Our findings of the presence of a dominant stricture and increased age of PSC diagnosis being risk factors for development of CCA have been previously reported in the literature [23, 24]. The increased association of CCA with colectomy has been demonstrated in other studies along with presence of IBD [25, 26], although the latter was not replicated in our findings. It is unclear the mechanisms that colectomy may confer increased risk of CCA, but this finding lends weight to the argument that the gut microbiome plays a pathogenetic role in disease development in PSC.

The reported transplant-free survival of 13 years in our Australian cohort is comparable to the largest international PSC cohort ever described—derived from 35 tertiary centers of over 7000 patients [19]. This also matched the estimated median transplant-free survival in transplant cohorts described by Boonstra et al. [20], of which the study also highlighted the greatly improved median transplant-free survival in the population-based cohort of 21.3 years in comparison to 13.2 years in the transplant center cohort. Increased age at PSC diagnosis, development of cirrhosis and CCA has also been found to be significant predictors of transplant-free survival in keeping with the current literature [21, 23, 27].

Another important observation from our study was that patients who are diagnosed at an older age do poorly compared to their same-aged counterparts in the general population. This effect is even seen in younger age groups, although it is more pronounced as patients are diagnosed at increasing age especially after 60 years. Overall, our findings do support the current literature that age of diagnosis has a significant impact on disease course [16, 19, 21], possibly related to an unique disease phenotype [23].

Our study highlights the difficulty and importance of diagnosing PSC early and suggests a role for screening higher risk individuals such as middle-aged males of European descent, particularly those with IBD. In addition, non-smokers may be at increased risk of developing PSC [28], including those with first-degree relatives with PSC [29]. Once the diagnosis of PSC is established, it is prudent to monitor for development of complications such as cirrhosis and CCA especially in older patients. Although there is lacking evidence on the benefit of surveillance of hepatobiliary malignancy in PSC, this is strongly recommended by international guidelines [9, 30] and screening (especially for CCA) may be warranted in all patients but perhaps more frequently in those with risk factors such as diagnosed at an older age, presence of a dominant stricture or history of colectomy.

### Strengths and limitations

Our study strengths include being the largest multicentre study of PSC in the Southern Hemisphere and long duration of patient follow-up. We also included comprehensive clinical endpoints such as the development of cirrhosis, malignancy, survival and need for LT. This was compared to the general population to derive the impact of PSC-related complications on overall morbidity and mortality. Identified limitations include our study being a retrospective analysis, as we acknowledge the real-world challenges of complete data collection and potential bias in case acquisition. Clinical delays with PSC diagnosis due to the typical asymptomatic presentation of PSC [1] may have affected results of the survival analysis. This is a limitation with most retrospective studies on PSC as we cannot accurately determine its subclinical course. However, our study reflects real-world data that correlates with other PSC cohorts as previously described. Although we did not exclude patients with other liver diseases, this contributed to less than 1% of the cohort and by including these patients allowed for a greater estimate of the epidemiology of PSC in Victoria.

We estimated the prevalence and incidence of a Victorian tertiary cohort of PSC patients in greater Melbourne; however, this does not reflect a true population-based result due to possible incomplete case acquisition and referrals from other states (Tasmania) to our Victorian liver transplant center contributing to a wider catchment population than expected. We also possibly missed out on a small subgroup of patients who may be managed privately or regionally that may have milder phenotypes of disease with improved outcomes. We acknowledge that some patients in our cohort were included in previous Australian studies [5, 6].

### Summary and conclusion

This study describes the demographics, clinical characteristics, and outcomes of the largest ever reported Australian cohort of PSC patients. We demonstrate a reduction in relative survival in patients diagnosed with older age with increased time from diagnosis, coupled with increased morbidity and mortality from development of CCA and cirrhosis. In addition, we found that presence of a dominant stricture and previous history of colectomy were risk factors for CCA.

Further prospective studies on PSC in Australia encompassing clinical data and serial prognostic markers are underway, which will allow us to further stratify high-risk patient groups and improve our understanding and management of PSC patients.

## Supplementary Information

Below is the link to the electronic supplementary material.

Supplementary file1 (DOCX 296 KB)

## Data Availability

The data that support the findings of this study are available on request from the corresponding author, NT.

## Abbreviations

ALP: Alkaline phosphatase
ALT: Alanine aminotransferase
AST: Aspartate aminotransferase
CA 19-9: Carbohydrate antigen 19-9
CCA: Cholangiocarcinoma
CI: Confidence interval
CRC: Colorectal carcinoma
HCC: Hepatocellular carcinoma
HR: Hazard ratio
IBD: Inflammatory bowel disease
IBDU: Inflammatory bowel disease—unclassified
INR: International normalized ratio
IQR: Interquartile range
LT: Liver transplantation
PSC: Primary sclerosing cholangitis
SIR: Standardized incidence ratio
SMR: Standardized mortality ratio
UDCA: Ursodeoxycholic acid

## Declarations

### Clinical trials registration

This study was not registered as a clinical trial.

### Animal research

This article does not contain any studies with human or animal subjects.
