# Supplementary material for: Epidemiology and outcomes of primary sclerosing cholangitis: an Australian multicentre retrospective cohort study
Source: Hepatol Int. 2022 Jun 3;16(5):1094–104. doi: 10.1007/s12072-022-10356-1 (PMC9525417; doi:10.1007/s12072-022-10356-1)
Supplement: Supplementary file 1 — Supplementary file1 (DOCX 296 KB) [file 12072_2022_10356_MOESM1_ESM.docx]

Supplementary table 1: Cumulative relative survival estimates with confidence intervals for the study population

| **Years after diagnosis** | **Age** | | | |
| --- | --- | --- | --- | --- |
|  | **-30** | **30-45** | **45-49** | **60-** |
| 0.0 - 2.0 | 1.00 (1.00 -1.00) | 1.00 (1.00 -1.00) | 0.99 (0.93-1.00) | 1.00 (0.88-1.03) |
| 2.0 - 4.0 | 1.00 (0.96-1.00) | 1.00 (1.00 -1.00) | 0.97 (0.91-1.00) | 0.89 (0.71-0.98) |
| 4.0 - 6.0 | 0.98 (0.95-1.00) | 0.97 (0.90-1.00) | 0.97 (0.90-1.00) | 0.80 (0.58-0.94) |
| 6.0 - 8.0 | 0.98 (0.93-0.99) | 0.95 (0.87-0.98) | 0.95 (0.87-0.99) | 0.71 (0.46-0.90) |
| 8.0 - 10.0 | 0.98 (0.94-1.00) | 0.95 (0.87-0.99) | 0.93 (0.83-0.99) | 0.75 (0.48-0.95) |
| 10.0 - 12.0 | 0.98 (0.94-1.00) | 0.92 (0.82-0.97) | 0.91 (0.79-0.97) | 0.49 (0.08-0.95) |
| 12.0 - 14.0 | 0.95 (0.88-0.98) | 0.91 (0.80-0.96) | 0.85 (0.71-0.94) |  |
| 14.0 - 16.0 | 0.94 (0.85-0.98) | 0.84 (0.71-0.92) | 0.84 (0.68-0.94) |  |
| 16.0 - 18.0 | 0.89 (0.78-0.95) | 0.85 (0.71-0.93) | 0.78 (0.60-0.91) |  |
| 18.0 - 20.0 | 0.87 (0.74-0.94) | 0.76 (0.59-0.87) | 0.80 (0.62-0.93) |  |
| 20.0 - 22.0 | 0.87 (0.74-0.94) | 0.77 (0.60-0.88) | 0.67 (0.44-0.85) |  |
| 22.0 - 24.0 | 0.83 (0.67-0.92) | 0.73 (0.55-0.86) | 0.63 (0.38-0.84) |  |
| 24.0 - 26.0 | 0.84 (0.67-0.93) | 0.74 (0.56-0.87) | 0.47 (0.21-0.74) |  |

Supplementary Table 2: Cox proportional hazards model for development of cholangiocarcinoma

|  | **Univariate** | | | **Multivariate** | | | |
| --- | --- | --- | --- | --- | --- | --- | --- |
| **Predictor** | **HR** | **95% CI** | **P-value** | **HR** | **95% CI** | **P-value** | |
| Male | 1.27 | 0.526-3.07 | 0.60 |  | | | |
| Cirrhosis | 0.59 | 0.258-1.33 | 0.20 |  |  | |  |
| Dominant stricture | **4.31** | **1.84-10.1** | **<0.001** | **3.33** | **1.40-7.88** | | **0.006** |
| Overlap (AIH) | 1.71 | 0.131-2.39 | 0.43 |  | | | |
| IBD | 3.19 | 0.747-13.6 | 0.12 |  |  | |  |
| UDCA | 1.47 | 0.583-3.71 | 0.41 |  | | | |
| Age at diagnosis | **1.03** | **1.01-1.06** | **0.009** | **1.03** | **1.00-1.05** | | **0.031** |
| ALP at diagnosis | 0.99 | 0.997-1 | 0.59 |  |  | |  |
| Bilirubin at diagnosis | 0.99 | 0.988-1.01 | 0.87 |  |  | |  |
| CA 19-9 at diagnosis | 0.99 | 0.937-1.04 | 0.62 |  |  | |  |
| Colectomy | **3.25** | **1.42-7.42** | **0.005** | **2.95** | **1.29-6.76** | | **0.011** |

AIH; autoimmune hepatitis, ALP; alkaline phosphatase, CA 19-9; carbohydrate antigen 19-9, CI; confidence interval, HR; hazard ratio, IBD; inflammatory bowel disease, UDCA; ursodeoxycholic acid

Supplementary Table 3: Cox proportional hazards model for transplant-free survival

|  | **Univariate** | | | | **Multivariate** | | | | |
| --- | --- | --- | --- | --- | --- | --- | --- | --- | --- |
| **Predictor** | **HR** | **95% CI** | **p-value** | **HR** | | **95% CI** | | **p-value** | |
| Male | 0.79 | 0.548-1.15 | 0.22 |  | | | | | |
| Cirrhosis | **5.72** | **3.50-9.34** | **<0.0001** | **6.49** | | | **2.90-14.6** | | **<0.0001** |
| Dominant stricture | **1.53** | **1.06-2.22** | **0.025** | 0.758 | | | 0.42-1.37 | | 0.356 |
| Small duct subtype | 0.46 | 0.188-1.12 | 0.088 |  | | | | | |
| Overlap (AIH) | 1.08 | 0.643-1.81 | 0.772 |  | | | | | |
| Cholangiocarcinoma | **2.56** | **1.58-4.14** | **<0.0001** | **3.03** | | | **1.28-7.15** | | **0.011** |
| Hepatocellular carcinoma | 1.91 | 0.777-4.69 | 0.16 |  | | | | | |
| Colorectal carcinoma | 0.82 | 0.336-2.02 | 0.67 |  | | | | | |
| IBD | **0.64** | **0.435-0.938** | **0.022** | 0.747 | | | 0.445-1.25 | | 0.270 |
| IBD Type  Crohn’s  IBDU | 0.85  1.44 | 0.507-1.41  0.35-5.92 | 0.52  0.61 |  | | | | | |
| UDCA | 1.17 | 0.788-1.73 | 0.44 |  | | | | | |
| Age at diagnosis | **1.02** | **1.01-1.03** | **<0.001** | **1.02** | | | **1.007-1.03** | | **0.0028** |
| ALP at diagnosis | **1.001** | **1.00-1.00** | **0.0075** | 1.000 | | | 1.00-1.00 | | 0.0887 |
| Bilirubin at diagnosis | **1.005** | **1-1.01** | **<0.001** | 1.003 | | | 0.999-1.01 | | 0.118 |

AIH; autoimmune hepatitis, ALP; alkaline phosphatase, CI; confidence interval, HR; hazard ratio, IBD; inflammatory bowel disease, IBDU; inflammatory bowel disease-unclassified, UDCA; ursodeoxycholic acid

**Supplementary Figure 1**

**
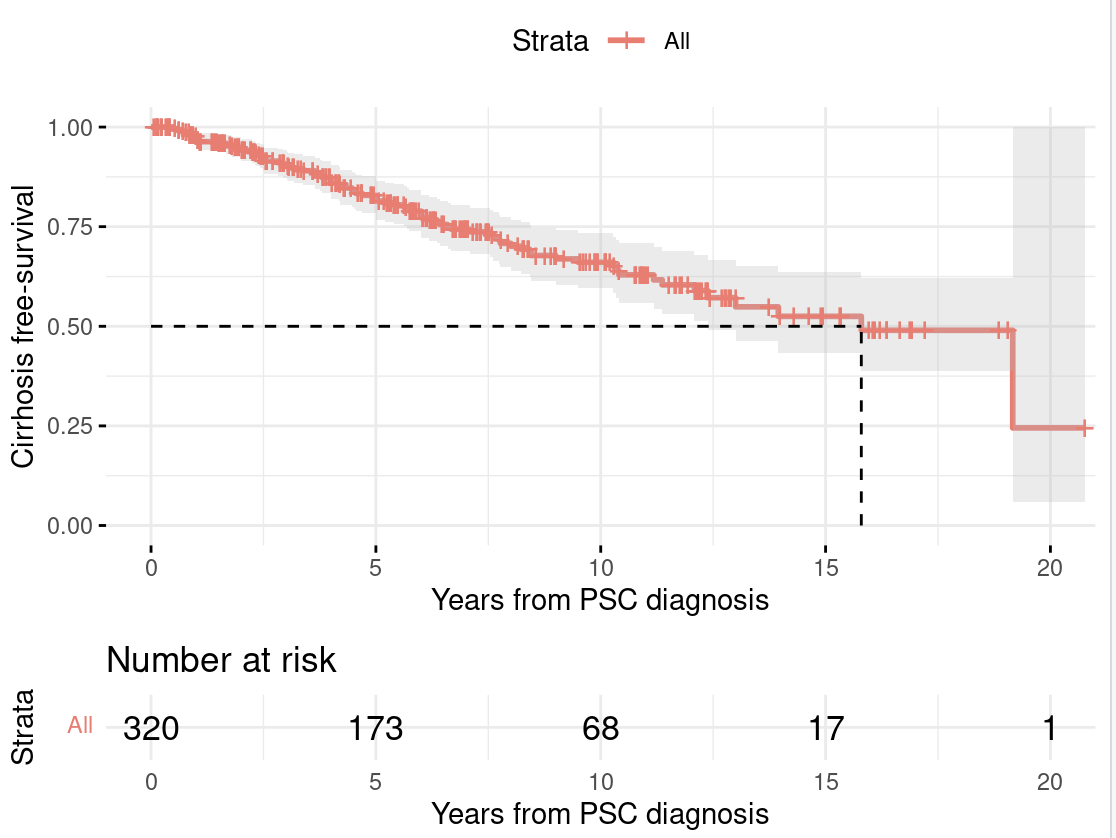
**

| **No. of Subject** | **Event** | **Censored** | **Median cirrhosis-free survival (95% CI)** | **5 years cirrhosis-free survival** | **10 years cirrhosis-free survival** | **20 years cirrhosis-free survival** |
| --- | --- | --- | --- | --- | --- | --- |
| 320 | 82 (25.6%) | 238 (74.4%) | 15.8 (12.4, NA) | 82.4% (77.9%, 87.3%) | 66.1% (59.4%, 73.4%) | 24.5% (6.0%, NA) |

**Supplementary Figure 2**

**Supplementary Figure 3**


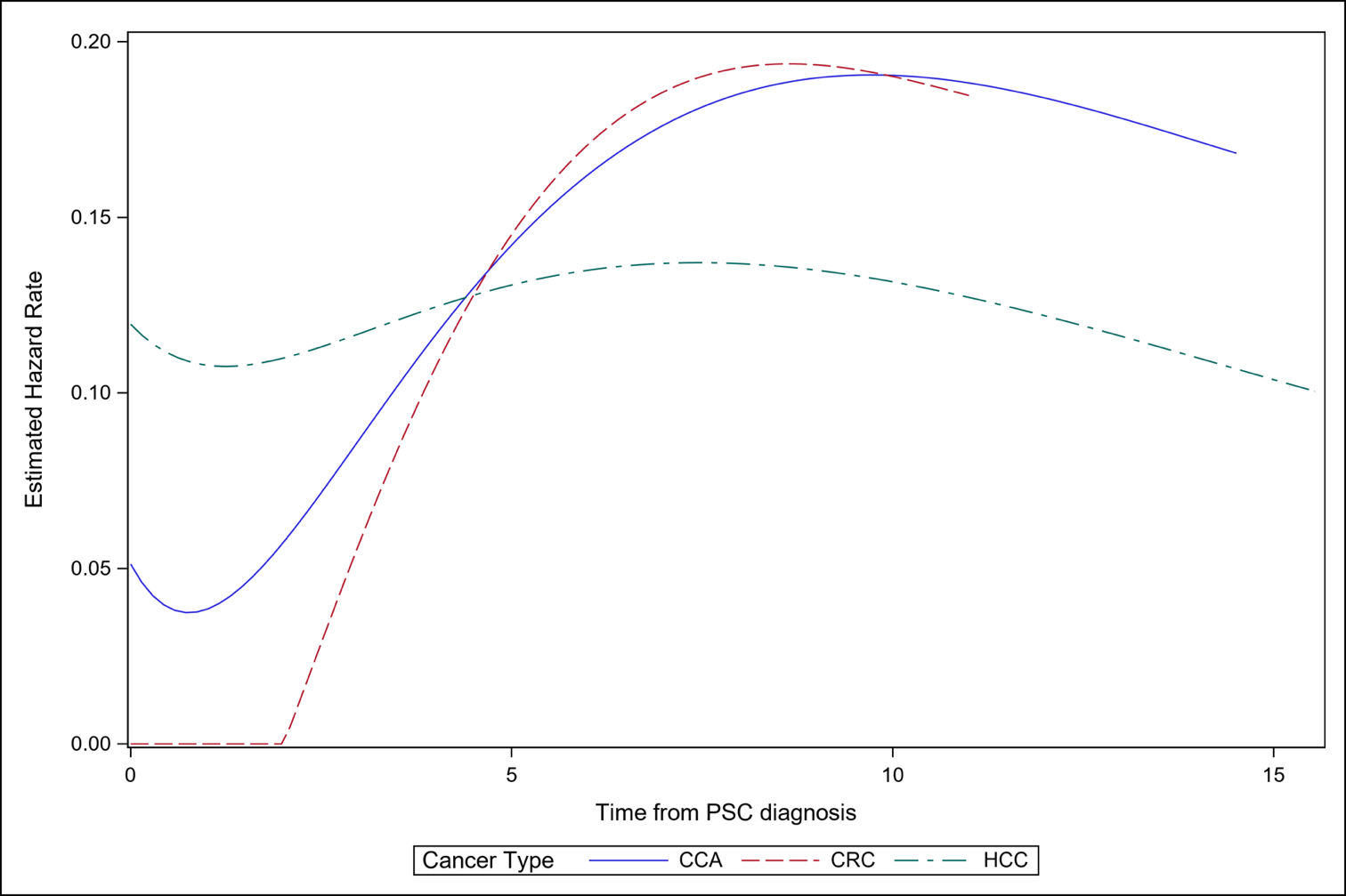


CCA; cholangiocarcinoma, CRC; colorectal carcinoma, HCC; hepatocellular carcinoma

**Supplementary Figure 4**

| **No. of Subject** | **Event** | **Censored** | **Median survival (95% CI)** |
| --- | --- | --- | --- |
| 24 | 18 (75%) | 6 (25%) | 0.6 (0.3, NA) |

**Supplementary Figure 5**

| **No. of Subject** | **Event** | **Censored** | **Median Survival (95% CI)** | **5 years Survival** | **10 years Survival** | **20 years Survival** |
| --- | --- | --- | --- | --- | --- | --- |
| 413 | 49 (12%) | 364 (88%) | NA (18.4, NA) | 95.2% (92.9%, 97.5%) | 89.6% (86.0%, 93.4%) | 60.3% (48.3%, 75.2%) |

**Supplementary Figure 6**

| **No. of Subject** | **Event** | **Censored** | **Median Survival (95% CI)** | **5 years Survival** | **10 years Survival** | **20 years Survival** |
| --- | --- | --- | --- | --- | --- | --- |
| 413 | 78 (18.9%) | 335 (81.1%) | NA (14.1, NA) | 93.1% (90.5%, 95.9%) | 77.7% (72.3%, 83.4%) | 50.1% (40.8%, 61.6%) |
